# Identification and Functional Analysis of the Mycophenolic Acid Gene Cluster of *Penicillium roqueforti*

**DOI:** 10.1371/journal.pone.0147047

**Published:** 2016-01-11

**Authors:** Abdiel Del-Cid, Carlos Gil-Durán, Inmaculada Vaca, Juan F. Rojas-Aedo, Ramón O. García-Rico, Gloria Levicán, Renato Chávez

**Affiliations:** 1 Departamento de Biología, Facultad de Química y Biología, Universidad de Santiago de Chile, Santiago, Chile; 2 Departamento de Química, Facultad de Ciencias, Universidad de Chile, Santiago, Chile; 3 GIMBIO Group, Department of Microbiology, Faculty of Basic Sciences, Universidad de Pamplona, Pamplona, Colombia; The University of Tokyo, JAPAN

## Abstract

The filamentous fungus *Penicillium roqueforti* is widely known as the ripening agent of blue-veined cheeses. Additionally, this fungus is able to produce several secondary metabolites, including the meroterpenoid compound mycophenolic acid (MPA). Cheeses ripened with *P*. *roqueforti* are usually contaminated with MPA. On the other hand, MPA is a commercially valuable immunosuppressant. However, to date the molecular basis of the production of MPA by *P*. *roqueforti* is still unknown. Using a bioinformatic approach, we have identified a genomic region of approximately 24.4 kbp containing a seven-gene cluster that may be involved in the MPA biosynthesis in *P*. *roqueforti*. Gene silencing of each of these seven genes (named *mpaA*, *mpaB*, *mpaC*, *mpaDE*, *mpaF*, *mpaG* and *mpaH*) resulted in dramatic reductions in MPA production, confirming that all of these genes are involved in the biosynthesis of the compound. Interestingly, the *mpaF* gene, originally described in *P*. *brevicompactum* as a MPA self-resistance gene, also exerts the same function in *P*. *roqueforti*, suggesting that this gene has a dual function in MPA metabolism. The knowledge of the biosynthetic pathway of MPA in *P*. *roqueforti* will be important for the future control of MPA contamination in cheeses and the improvement of MPA production for commercial purposes.

## Introduction

Mycophenolic acid (MPA) is one of the main metabolites produced by fungi of the genus *Penicillium* [1, 2]. From a chemical point of view, MPA is a meroterpenoid composed of a phthalide moiety substituted by a terpenoid side chain. Since its discovery and over the years, MPA has been shown to have antibacterial, antitumoral and antiviral properties [3]. However, by far the most important clinical application of this compound is its use as an immunosuppressant in transplantation patients, and MPA derivatives are currently commercially available for this purpose [3, 4].

The molecular basis of the biosynthesis of MPA was unknown until recently. The genomic cluster that may be responsible for MPA biosynthesis has been identified in *P*. *brevicompactum* [4–6]. In this fungus, the cluster consists of seven genes named *mpaA* (encoding a putative prenyltransferase), *mpaB* (encoding a protein with unknown function), *mpaC* (encoding a polyketide synthase), *mpaDE* (encoding a natural fusion of a cytochrome P450 domain and a hydrolase domain), *mpaF* (encoding a protein with high similarity to inosine-5´-monophosphate dehydrogenase, IMPDH), *mpaG* (encoding an O-methyltransferase) and *mpaH* (encoding an oxidative cleavage enzyme) [4–6].

Thus far, by using different techniques, only three out of the seven *mpa* genes from *P*. *brevicompactum* have been experimentally shown to be involved in MPA biosynthesis. The *mpaC* gene was characterized by gene disruption. A mutant strain lacking this gene lost its ability to produce MPA [4]. MpaC catalyzes the formation of 5-methylorsellinic acid (5-MOA), which is the first step in MPA biosynthesis [4]. Regarding *mpaDE*, this gene was biochemically characterized *in vivo* by heterologous expression in a strain of *Aspergillus nidulans* that expresses *mpaC* and is able to produce 5-MOA [5]. This biochemical characterization allowed the reconstitution of the second step in MPA biosynthesis: the consecutive transformation of 5-MOA to 4,6-dihydroxy-2-(hydroxymethyl)-3-methylbenzoic acid and 5,7-dihydroxy-4-methylphthalide (DHMP) by the bifunctional enzyme MpaDE [5]. Finally, MpaG, the putative O-methyl transferase, was biochemically characterized *in vitro*. The *mpaG* gene from *P*. *brevicompactum* was overexpressed in *E*. *coli* and the recombinant MpaG protein was purified and used to reconstitute its activity with pure substrates [6]. The results indicate that MpaG catalyzes the methylation of demethylmycophenolic acid (DMMPA) to produce MPA, the last step in the biosynthesis of this compound [6]. To the best of our knowledge, the role of the other four *mpa* genes in the biosynthesis of MPA has not yet been experimentally addressed.

*Penicillium roqueforti* is a filamentous fungus that is very important to the food industry. This fungus is responsible in large measure for the organoleptic properties of several types of blue-veined cheeses from different countries, such as Roquefort, Stilton, Danablu and Cabrales. In addition, this fungus is an active producer of several secondary metabolites, including MPA [2, 7, 8]. The presence of MPA in different types of cheeses ripened with *P*. *roqueforti* has been demonstrated and has been a constant concern [9–14]. On the other hand, recent experiments suggest that *P*. *roqueforti* strains submitted to random-mutagenesis by UV and gamma irradiation may be suitable for the commercial production of MPA [15]. Therefore, knowledge of the molecular basis underlying to the biosynthesis of MPA in *P*. *roqueforti* would be very useful for both the control of MPA contamination in cheeses and the potential commercial production of MPA. However, to date the biosynthetic pathway of MPA in *P*. *roqueforti* remains totally unknown.

In this work, we identified a genomic region of approximately 24.4 kbp containing a seven-gene cluster (the *mpa* cluster) that may be responsible for the MPA biosynthesis in *P*. *roqueforti*. Of greater interest, the functional analysis of these genes suggests that all of the seven genes are necessary for the production of MPA by the fungus.

## Materials and Methods

### Fungal strains and general molecular techniques

The wild-type strain of *P*. *roqueforti* CECT 2905 (ATCC 10110), originally isolated from a blue cheese sample, was used in this work. *Penicillium roqueforti* transformants were obtained by protoplast transformation of strain CECT 2905, as described below. Potato dextrose agar (PDA; Merck, Germany) was used for the routine growth of all the strains.

The protocol described by Gil-Duran et al. [16] was used for DNA isolation. For total RNA purification, the strains were grown on YES agar (Bacto Yeast Extract (Difco, USA) 20 g/l, sucrose (Merck, Germany, biochemical grade) 150 g/l and Bacto Agar (Difco, USA) 20 g/l) for 7 days at 28°C. Total RNA was isolated exactly as described previously [16]. RT-PCR experiments were performed as described by Ravanal et al. [17]. RACE-PCR experiments were performed with the FirstChoice RLM-RACE Kit (Invitrogen, USA) according to the manufacturer`s protocol.

### Bioinformatic identification and characterization of the *mpa* cluster of *P*. *roqueforti*

The highly conserved MpaF protein, encoded by the *mpaF* gene from *P*. *brevicompactum*, was used to scan the whole draft genome of *P*. *roqueforti* [18] by tBlastN. Two contigs (GenBank accessions CBMR010000200 and CBMR010000309) containing ORFs encoding proteins with significant similarity to MpaF were identified and downloaded. The vicinities of each of these ORFs were then manually analyzed by BlastX, BlastP and BlastN. All Blast searches were performed using the online web interface at http://blast.ncbi.nlm.nih.gov. Multiple alignments of deduced proteins were performed using Clustal Omega at http://www.ebi.ac.uk/Tools/msa/clustalo/.

### Construction of plasmids to silence the seven genes of the MPA gene cluster

RNA-mediated gene silencing technology has been successfully used to silence genes in *P*. *roqueforti* [16, 19]. Here we used the same experimental approach. Plasmid pJL43-RNAi [20] was used to generate seven constructs (S1 Fig) as follows: pJL43-RNAi was digested with *Nco*I. In parallel, a small fragment of each gene from the *mpa* cluster was amplified with suitable primers and was also digested with *Nco*I. Finally, each digested fragment was ligated into pJL43-RNAi, thus giving rise to the plasmids pJL-RNAi-mpaA, pJL-RNAi-mpaB, pJL-RNAi-mpaC, pJL-RNAi-mpaDE, pJL-RNAi-mpaF, pJL-RNAi-mpaG and pJL-RNAi-mpaH, which were used to transform *P*. *roqueforti* (see below). These plasmids contain the respective DNA fragment flanked by two promoters in opposite directions (S1 Fig), which generate double-stranded RNA molecules (dsRNAs). These dsRNAs activate the fungal RNA-silencing machinery, resulting in the degradation of target mRNAs [20]. S1 Table shows the primer sequences and the size of the fragment ligated in each construct.

### Transformation of *P*. *roqueforti*

*P*. *roqueforti* transformants were obtained by introducing the genetic constructs described above into strain CECT 2905 by protoplast transformation. Protoplast obtainment, transformation, selection of transformants on Czapek-sorbitol medium containing phleomycin, and obtainment of homokaryotic strains were carried out exactly as described before [16].

### Quantitative reverse-transcriptase polymerase chain reaction (qRT-PCR) analysis

The silencing level of each gene in the transformants was analyzed by qRT-PCR as described previously [16]. Total RNA was quantified in a MultiSkan GO quantification system (Thermo Scientific, Germany). Two μg were used to synthesize cDNA using RevertAid Reverse Transcriptase (Thermo Scientific, Germany). Each qRT-PCR reaction (20 μl) contained 10 μl of KAPA SYBR Fast qRT-PCR Master Mix 2x (Kapa Biosystems, USA), 0.4 μl of each primer (at a concentration of 10 μM each), 0.4 μl de 50x ROX High/Low, 6.8 μl of water and 2 μl of cDNA. qRT-PCR reactions were carried out using the StepOne Real-Time PCR System (Applied Biosystems, USA). Amplification conditions were as follows: 20 s at 95°C and 40 cycles of 3 s at 95°C and 30 s at 50°C. Three replicates were performed for each analysis and suitable negative controls were included. Relative gene expression values were determined by the comparative Ct (ΔΔCt) method using β-tubulin gene expression as a normalization control. S2 Table describes the sequence of the primer sets used in qRT-PCR. All exhibited suitable efficiencies as required for the comparative Ct (ΔΔCt) method (S3 Table).

### Extraction and HPLC analysis of MPA

Each fungal strain was grown on YES agar (see the composition above) for 7 days at 28°C. For each strain the mycelium from three Petri dishes was scraped off the agar. After that, the remaining agar was triturated. Both the mycelium and the triturated agar were extracted separately according to the method described by O´Brien et al. [21], with some modifications. Each sample (mycelium or agar) was extracted overnight with 50 ml of an ethyl acetate: dichloromethane: methanol (3:2:1) mixture containing formic acid (1%). The mixtures were then sonicated for 30 minutes, filtered through a 0.45 μm Millex-HV hydrophilic PVDF syringe filter (Merck Millipore), and evaporated to dryness in a rotary evaporator. The extracts were resuspended in 500 μl of methanol (HPLC grade) just before HPLC analysis. HPLC analysis was performed on a Waters 1525 HPLC system (Waters, Ireland) equipped with a Waters 1525 Binary HPLC pump and a Waters 2996 Photodiode Array (PDA) Detector, using the chromatographic method described by Nielsen et al. [22] with some modifications. Briefly, 20 μl of the methanol suspension described before were injected into a 4.6 x 250 mm (5 μm) SunFire C18 column (Waters, Ireland). The column was held at 35°C. A solvent gradient with water and acetonitrile (both acidified with 0.02% trifluoroacetic acid) at a flow rate of 1.2 ml/min was used. The gradient began with 15% acetonitrile and was increased to 68% acetonitrile after 25 minutes. The acetonitrile was then increased to 100% within 2 min. This percentage remained constant for 5 minutes before returning to 15% acetonitrile. MPA was detected by UV absorption at 300 nm and quantified using the pure compound as standard (purchased from Santa Cruz Biotechnology, Dallas, TX). MPA showed a retention time of 18.25 min. For each strain used, the quantity of MPA was normalized by the dry weight of the fungal mycelia. For this purpose, and after the extraction with ethyl acetate/dichloromethane/methanol, the mycelia from each sample were dried according to García-Rico et al. [23].

### Analysis of the MPA resistance in *P*. *roqueforti* strains

MPA treatment of the fungi was performed by the spot assay method described by Hansen et al. [24]. Briefly, spores from *P*. *roqueforti* strains were harvested, and a suspension of 1x10^6^ spores/ml was obtained. From this initial suspension, 10-fold dilution series were performed on freshly made CYA-plates containing 100 μg MPA/ml (Santa Cruz Biotechnology, Dallas, TX). Plates were incubated for 9 days at 28°C.

## Results and Discussion

### Identification of the MPA gene cluster in the *Penicillium roqueforti* genome

To find orthologs of the MpaF protein from *P*. *brevicompactum*, we scanned the whole genome of *P*. *roqueforti* (see Methods). We found two protein orthologs in the *P*. *roqueforti* genome (Genbank accession numbers CDM33495 and CDM36723), which were previously annotated as putative IMPDHs [18]. CDM33495 is a 546-amino acid protein, encoded by ORF Proq03g044510, whereas CDM36723 is a 526-amino acid protein, encoded by ORF Proq05g069770. It has been stated that members of the *Penicillium* subgenus *Penicillium* usually contain two IMPDH-like proteins, one corresponding to the true IMPDH enzyme and the other corresponding to MpaF, a protein with high similarity to IMPDH [25].

**Fig 1:**
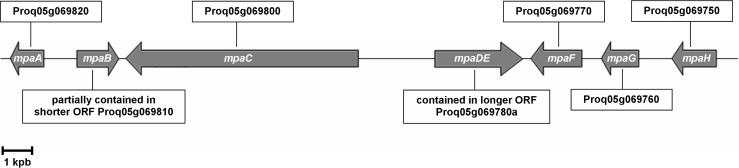
Schematic organization of the MPA biosynthetic gene cluster in *P*. *roqueforti*. The arrows indicate the genes and the direction of their transcription. The correspondence of each *mpa* gene with their respective ORFs previously annotated in the draft genome of *P*. *roqueforti* [18] is indicated in the boxes. According to our analysis, *mpaB* should be longer than ORF Proq05g069810, so this ORF would partially contain *mpaB*. In contrast, Proq05g069780a is longer than the *mpaDE* gene predicted by us.

The two contigs containing these ORFs were downloaded and they were scanned when searching for the rest of the putative MPA biosynthetic genes. Analysis of the vicinity of Proq03g044510 did not yielded positive results (data not shown). However, analysis of the vicinity of Proq05g069770 revealed the presence of six other putative genes forming a cluster with identical gene organization (Fig 1) and high similarity (73% average nucleotide identity between coding regions) to the *mpa* gene cluster from *P*. *brevicompactum* [6]. In accordance with the nomenclature previously used for *P*. *brevicompatum*, we named these genes *mpaA*, *mpaB*, *mpaC*, *mpaDE*, *mpaF*, *mpaG* and *mpaH* (Fig 1). Our independent delimitation of the coding sequences of five of these genes exactly matched the original delimitation of ORFs performed on the draft genome of *P*. *roqueforti* [18], but we found discrepancies in the delimitation of two genes. The original annotation of ORF Proq05g069810 (belonging to *mpaB*) predicts a coding sequence of 949 nt containing two exons. However, our analysis predicts that the gene spans a larger region (1,397 nt) and contains an additional exon at the 3´ end (Fig 1, S2 Fig). Also, the original annotation of ORF Proq05g069780a (belonging to *mpaDE*) predicts a coding sequence of 3,710 nt containing 9 exons, whereas our independent analysis indicates that the gene is shorter (2,929 nt; 788 nt in excess at the 5´ end) and contains 7 exons (Fig 1, S3 Fig). To experimentally confirm these predictions, we used RACE-PCR methodology. The results confirmed the delimitation of ORFs predicted by us (S4 Fig). These findings should be considered for future hypothetical heterologous expressions of *mpaB* and *mpaDE*. Annotating a eukaryotic genome is a challenging task, and despite the advance in genome annotation pipelines, incidents such as genes of incorrect size or misannotated intron/exon junctions are unavoidable [26].

Taking into account the conserved genetic organization of the *mpa* cluster and the high identity between the coding regions of the *mpa* genes, few differences in the MPA production levels between *P*. *roqueforti* and *P*. *brevicompactum* would be expected. However, it has been described that whereas *P*. *brevicompactum* produces MPA consistently and in high amounts, *P*. *roqueforti* is an inconsistent producer and its levels of production of MPA are lower compared with *P*. *brevicompactum* [2, 10, 27]. Thus, we tested if the comparison of non-coding regions between the *mpa* clusters could shed lights over these differences. Our results indicated that the similarity between the intergenic regions is extremely low (S5 Fig), suggesting that the differences in MPA production between both fungi could be due to differences in the promoter sequences, which in turn would produce putative differences in the expression patterns of their *mpa* genes. In the future, this hypothesis should be tested by the comparative analysis of the expression of the *mpa* genes in both fungi.

The deduced proteins encoded by the *mpa* genes from *P*. *roqueforti* were analyzed, and the results are summarized in Table 1. As a consequence of the discrepancies in delimiting *mpaB* and *mpaDE*, the sizes of the deduced proteins MpaB and MpaDE we obtained do not match the original annotations described in Genbank. According to our analysis, MpaB should be larger than the original annotation (CDM36727; Table 1, S2 Fig). Conversely, the deduced MpaDE protein should be shorter than originally deduced (CDM36724; Table 1, S3 Fig).

**Table 1:** Analysis of the deduced proteins of the *mpa* cluster of *P*. *roqueforti*.

				Identity with *P*. *brevicompactum* orthologs	
Protein name	Protein accession number in Genbank	Size of the deduced protein (aminoacids)	Putative function in MPA biosynthesis^c^	Protein accession number in Genbank	Identity (%)
MpaA	CDM36728	325	Prenyltransferase	AJG44379	80
MpaB	CDM36727	427^a^	Unknown (similarity with dephospho-CoA kinases)	AJG44380	80
MpaC	CDM36726	2,477	Polyketide synthase (PKS)	ADY00130	72
MpaDE	CDM36724	852^b^	Bifunctional cytochrome P450-hydrolase	AJG44382	79
MpaF	CDM36723	526	IMPDH-like protein; self-resistance to MPA	ADY00133	92
MpaG	CDM36722	398	O-methyl transferase	AJG44384	77
MpaH	CDM36721	433	Oxidative cleavage enzyme	ADY00135	82

^a^ In the original annotation of the genome draft of *P*. *roqueforti* [18], this protein is shorter than the predicted by our independent analyses.

^b^ In the original annotation of the genome draft of *P*. *roqueforti* [18], this protein is longer than the predicted by our independent analyses.

^c^ Putative functions of the orthologous proteins of *P*. *brevicompactum* [4–6, 25].

### RNA-mediated silencing of the seven genes of the MPA gene cluster of *Penicillium roqueforti*

**Fig 2:**
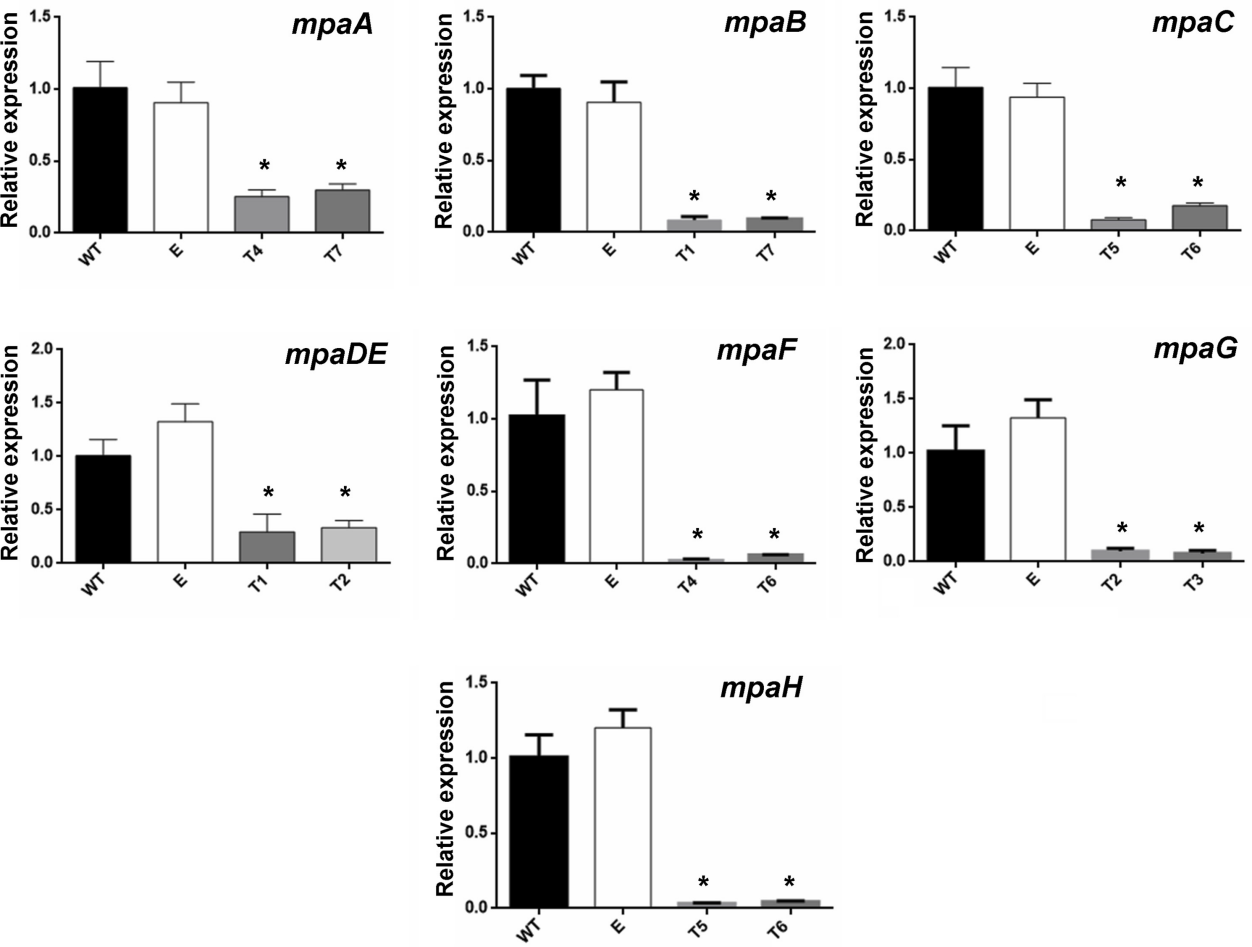
qRT-PCR analysis of the expression of *mpa* genes in RNAi-silenced transformants of *P*. *roqueforti*. The RNAi-silenced transformants selected were T4 and T7 for *mpaA*, T1 and T7 for *mpaB*, T5 and T6 for *mpaC*, T1 and T2 for *mpaDE*, T4 and T6 for *mpaF*, T2 and T3 for *mpaG*, and T5 and T6 for *mpaH*. Total RNA extractions and qRT-PCR experiments were conducted as described in Materials and Methods. Wild-type *P*. *roqueforti* CECT 2905 (WT) and *P*. *roqueforti* CECT 2905 containing empty pJL43-RNAi vector (E) were used as controls. Error bars represent the standard deviation of three replicates in three different experiments. The differences were considered statistically significant at *P* < 0.05 (*) using Student’s *t*-test.

Thus far, three of the seven genes from the *mpa* cluster (*mpaC*, *mpaDE* and *mpaG*) have been experimentally involved in the biosynthesis of MPA in *P*. *brevicompactum* (see Introduction). Whether the remaining genes have roles in MPA biosynthesis is currently unknown. To test the participation of all the genes from the *P*. *roqueforti mpa* gene cluster in MPA biosynthesis, we employed RNA-mediated gene-silencing technology. Seven suitable plasmids were constructed and used to transform *P*. *roqueforti* CECT 2905. Approximately 40 phleomycin-resistant transformants were obtained after each transformation. Several of these were randomly selected and subjected to preliminary RT-PCR analysis (data not shown). Those transformants showing drastic reductions in the levels of the gene transcripts were selected for further quantification of the amount of down-regulation using qRT-PCR (Fig 2). Depending on the gene, the transformants selected exhibited between 2.99- and 44.7- fold decreases in transcripts compared with the wild-type strain of *P*. *roqueforti* (Fig 2), confirming the successful knockdown of the expression of all the genes of the *mpa* cluster. Additionally, the presence of the full silencing cassette in each of these transformants was confirmed (S6 Fig). These transformants were used for further analysis.

### The silencing of *mpaA*, *mpaB*, *mpaC*, *mpaDE*, *mpaG* and *mpaH* decreased the production of MPA in *Penicillium roqueforti*

**Fig 3:**
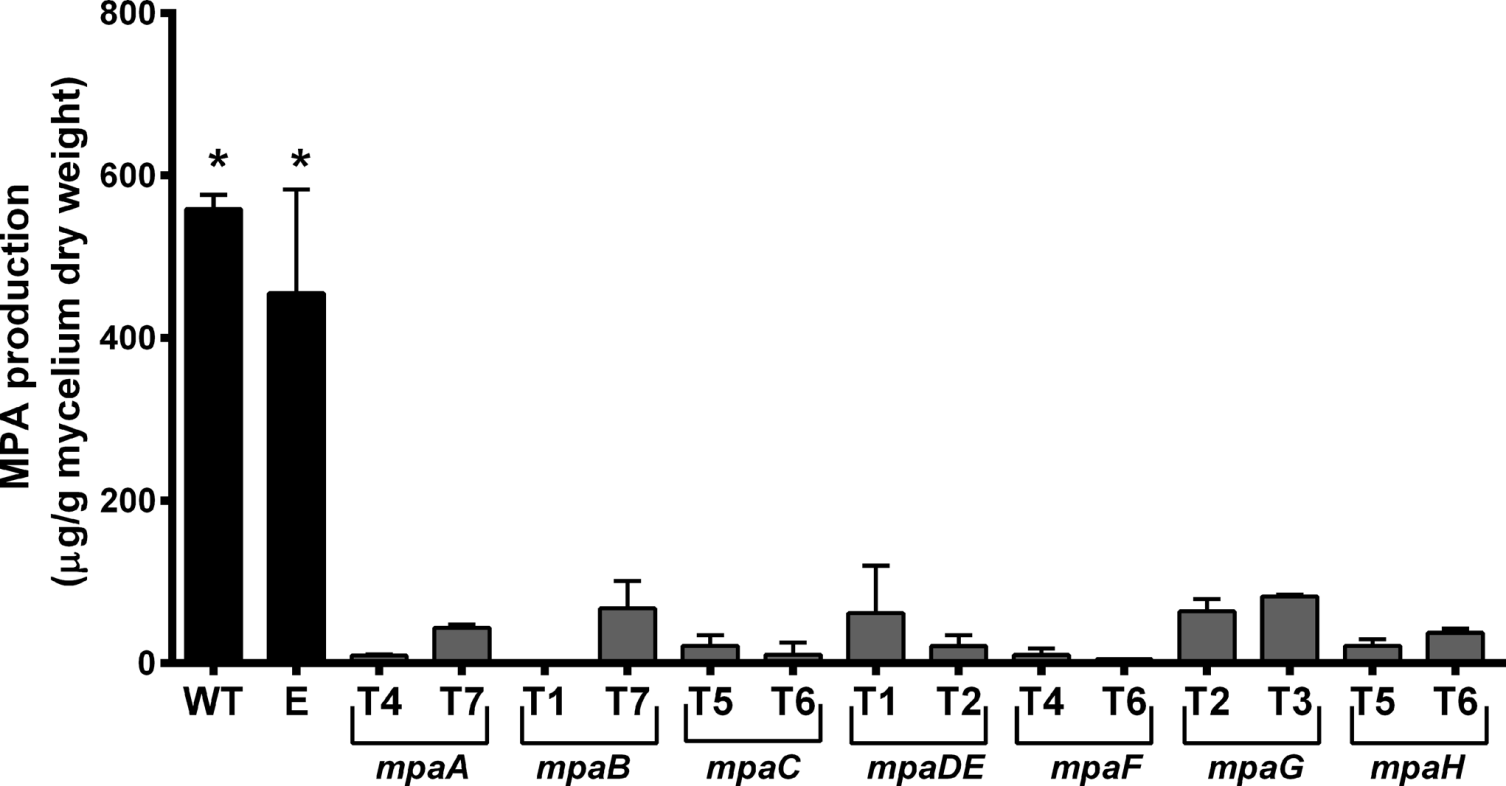
Production of MPA by *P*. *roqueforti* (WT) and RNAi-silenced transformants. Transformants are the same as described in Fig 2. Metabolites were extracted from mycelium and quantified as described in Materials and Methods. In each case, the quantity of MPA was normalized by the dry weight of the fungal mycelia extracted. Error bars represent the standard deviation of three replicates in three independent experiments. Statistical analysis using Student’s *t*-test (P < 0.05) indicates significant differences between the production of MPA by the wild-type strain (*) and the transformants. Please note that MPA production of *P*. *roqueforti* containing empty pJL43-RNAi vector was statistically indistinguishable from the wild-type strain. When MPA was extracted from agar, the results were similar (S8 Fig).

Once silencing of the genes in the transformants was confirmed (see above), the production of MPA was analyzed by HPLC. The gene silencing of each of the seven genes resulted in dramatic reductions in MPA production (Fig 3, S7 Fig) confirming that all of them are involved in the biosynthesis of the compound. Interestingly, we did not found significant differences in the level of MPA extracted from the mycelium (representing intracellular and mycelium associated MPA) and the agar (representing extracellular MPA) (S8 Fig) suggesting that none of these genes is involved in the secretion of MPA to the medium. In the following paragraphs we discuss the results in detail for each gene except for *mpaF*, which is discussed separately below.

Silencing of the *mpaA* gene in *P*. *roqueforti* drastically reduced the production of MPA. Transformants were able to produce between 1.9 and 8.2% of the MPA produced by the wild-type strain (Fig 3). Until now, the participation of *mpaA* in MPA biosynthesis had not been experimentally demonstrated. Our results are the first confirmation of the participation of this gene in the production of this secondary metabolite.

**Fig 4:**
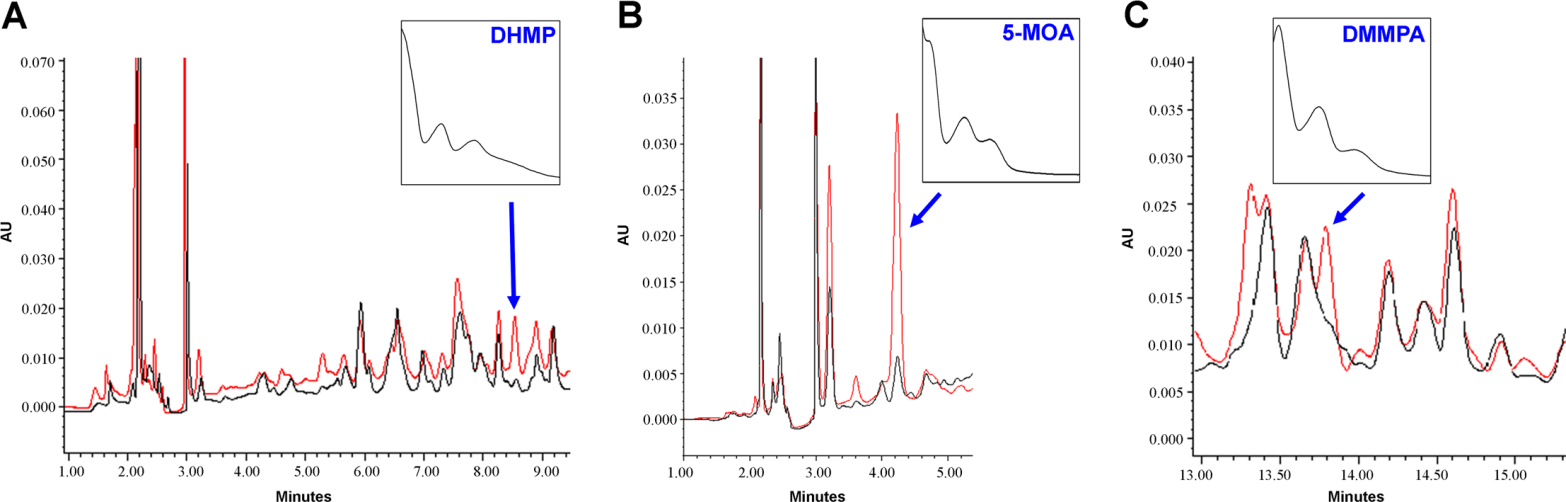
HPLC analysis of the known intermediates of the MPA biosynthesis pathway in RNAi-silenced transformants of *P*. *roqueforti*. (A) Accumulation of DHMP in the *mpaA* silenced transformant T7. (B) Accumulation of 5-MOA in the *mpaDE* silenced transformant T2. (C) Accumulation of DMMPA in the *mpaG* silenced transformant T3. The HPLC trace chromatograms (300 nm) of the transformants are shown as red line, whereas the trace chromatogram (300 nm) of the wild-type strain (control) is shown as black line. In each panel, the peak representing the accumulated compound is indicated by an arrow. The identity of each peak was assigned based on the expected retention time and its UV absorption spectrum (shown in the inset of each panel). The wavelengths of each UV absorption maximum (nm) are 216, 257 and 296 for DHMP; 217, 260 and 296 for 5-MOA; 216, 258 and 304 for DMMPA. These values are in agreement with those described previously [28, 29]. AU: Absorbance units.

MpaA is a protein with high similarity to prenyltransferases (Table 1). In *P*. *brevicompactum*, it has been hypothesized that, during MPA biosynthesis, MpaA catalyzes the addition of a farnesyl moiety to the phthalide intermediate DHMP [4]. Accordingly, the accumulation of DHMP should be observed in *mpaA*-silenced transformants. The analysis of the HPLC chromatograms allowed the identification of a peak that accumulates in *mpaA*-silenced transformants, whose UV spectrum agrees well with the previously published UV spectrum of DHMP [28] (Fig 4). This result supports the previously suggested role for MpaA in the biosynthesis of MPA.

Like *mpaA*, the *mpaB* gene has not been experimentally studied thus far. Its deduced protein (MpaB) has high similarity to the dephospho-CoA kinases (Table 1), but a hypothetical function has not yet been assigned. Thus, its implication in MPA production is unknown. Our results indicate that the silencing of *mpaB* in *P*. *roqueforti* drastically reduced the production of MPA (between 0.7 and 11.3% of the MPA produced by the wild-type strain; Fig 3), experimentally confirming for the first time the participation of *mpaB* in the production of MPA. Further work is necessary to establish the exact role of this protein in MPA biosynthesis.

In *P*. *brevicompactum*, *mpaC* was previously disrupted and the mutant strain lacking this gene does not produce MPA [4]. Our results also confirmed the involvement of this gene in MPA biosynthesis in *P*. *roqueforti*. Transformants with attenuated levels of *mpaC* transcripts produced between 2.1 and 3.9% of the MPA produced by the wild-type strain (Fig 3). In *P*. *brevicompactum*, *mpaC* encodes for a PKS which catalyzes the formation of 5-MOA from acetyl-CoA, malonyl-CoA, and S-adenosyl methionine [4]. This protein has a high similarity to MpaC from *P*. *roqueforti* (Table 1). In addition, when the domain structures of both MpaCs were compared, they were shown to be similar (data not shown). Experimentally, we observed the accumulation of 5-MOA in *mpaDE*-silenced transformants (Fig 4; see below). All together, these data suggests that MpaC may catalyze the same reaction in *P*. *roqueforti*.

As in *P*. *brevicompactum*, *mpaDE* in *P*. *roqueforti* encodes for a natural fusion of a cytochrome P450 and a hydrolase (Table 1). The gene *mpaDE* from *P*. *brevicompactum* was biochemically characterized by heterologous expression in *Aspergillus nidulans*, a non-producer of MPA [5], but we have no genetic evidence of its role in any producer organism itself. Our results provide this evidence for *P*. *roqueforti*, thus complementing the biochemical evidence previously obtained. We found that *P*. *roqueforti* transformants with attenuated levels of *mpaDE* transcripts produce between 3.7 and 10.3% of the MPA produced by the wild-type strain (Fig 3), confirming that this gene is necessary to the *in vivo* production of MPA by *P*. *roqueforti*. In addition, we observed the accumulation of 5-MOA in *mpaDE*-silenced transformants (Fig 4), suggesting that as its ortholog in *P*. *brevicompactum*, in *P*. *roqueforti* MpaDE catalyzes the transformation of 5-MOA to DHMP.

The *mpaG* gene encodes for a putative O-methyl transferase (Table 1). In *P*. *brevicompactum*, this gene was biochemically characterized *in vitro* and may catalyze the last step in the biosynthesis of MPA [6]; again, we have no genetic evidence of its role in any producer organism itself. In our case, we found that *P*. *roqueforti* transformants with attenuated levels of *mpaG* transcripts produce between 10.7 and 13.6% the MPA produced by the wild-type strain (Fig 3), thus experimentally confirming the participation of this gene in the *in vivo* production of MPA by *P*. *roqueforti*. In addition, the accumulation of DMMPA (the precursor of MPA) was observed in *mpaG*-silenced transformants (Fig 4). This suggests that as in *P*. *brevicompactum*, in *P*. *roqueforti* MpaG catalyzes the transformation of DMMPA to MPA.

Regueira et al. [4] suggested that MpaH would be involved in the cleavage of the farnesyl chain of the hypothetical farnesylated phthalide intermediate produced by MpaA. However, the participation of *mpaH* in MPA biosynthesis has not yet been experimentally demonstrated. In *P*. *roqueforti*, we observed that the silencing of *mpaH* drastically reduced the production of MPA (between 3.8 and 6.4% of the MPA produced by the wild-type strain; Fig 3). These results suggest that this gene is necessary for the production of MPA and represent the first confirmation of its participation in this metabolic pathway.

### The silencing of *mpaF* increased the sensitivity of *P*. *roqueforti* towards MPA and decreased MPA production by the fungus

**Fig 5:**
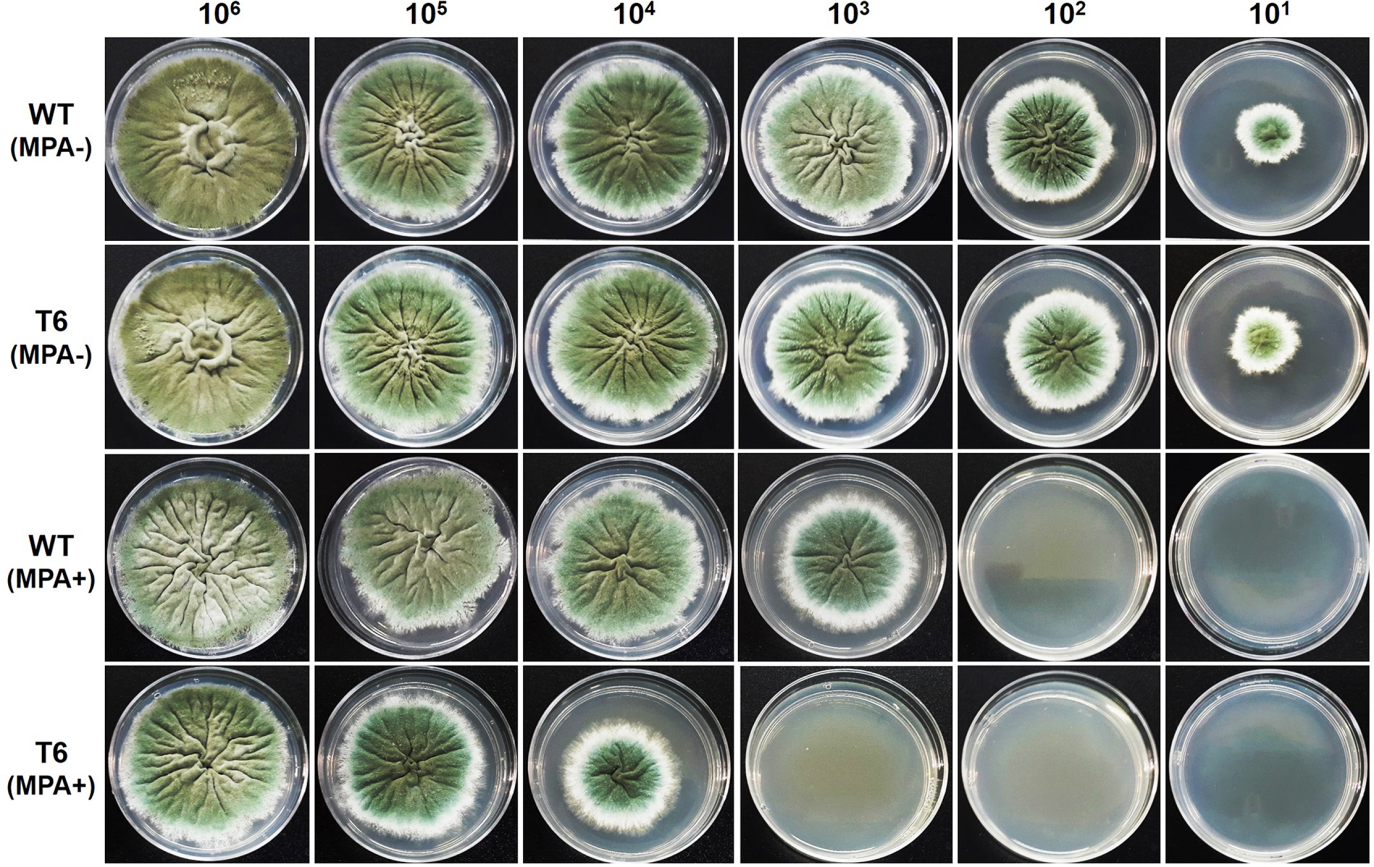
Sensitivity towards MPA of transformant T6, with attenuated expression of *mpaF*, and wild-type *P*. *roqueforti*. Ten-fold serial dilutions (10^6^ to 10) of spores from T6 and the wild-type strain (WT) were spotted on CYA plates with (+MPA) or without (-MPA) 100 μg MPA/ml. Please note the reduction in the germination of spores of transformant T6 in presence of MPA, especially evident at dilutions 10^4^ and 10^3^, compared with the wild-type strain.

The *mpaF* gene encodes for a protein with high similarity to IMPDH. IMPDH catalyzes the transformation of inosine-5'-monophosphate to xanthosine 5'-monophosphate, which is the rate-limiting reaction of *de novo* GTP biosynthesis [30]. Interestingly, IMPDH is inhibited by MPA [30]. These observations suggested a role of *mpaF* in MPA self-resistance. It was experimentally observed that the heterologous expression of *mpaF* from *P*. *brevicompactum* in *Aspergillus nidulans* (a non-producer of MPA) increased MPA resistance in the recipient fungus [24, 25]. According to these data, the attenuation of *mpaF* should decrease the resistance of *P*. *roqueforti* to MPA. The spot assays shown in Fig 5 indicate that this prediction was correct. The germination of serial dilutions of *P*. *roqueforti* spores is reduced by MPA, but this effect is clearly most significant for the strain with attenuated *mpaF* expression. These results are in agreement with previous observations suggesting that the sensitivity of *P*. *roqueforti* towards MPA is related to the expression of the *mpaF* gene.

Fungi have genes located in secondary metabolite clusters that are involved in the self-protection against specific metabolites and also have roles in their production. For example, in *Fusarium sporotrichioides* the disruption of the TRI12 gene results in reduced levels of trichothecene production and an enhanced sensitivity of the mutants to this metabolite [31]. In the gliotoxin-producing fungus *Aspergillus fumigatus*, the disruption of *gliT* gene produces an analogous effect: a reduced production of gliotoxin and sensitization of the mutants towards the compound [32]. Remarkably, the putative implication of *mpaF* in the production of MPA has never been tested. Therefore, we addressed the question of whether *mpaF* was also implied in the production of MPA. Our results indicate that the silencing of *mpaF* gene in *P*. *roqueforti* drastically reduced the production of MPA. Thus, transformants T4 and T6 produced between 1.1 and 2% the MPA produced by the wild-type strain (Fig 3). Since no phenotypic differences in the growth of these strains were observed during the incubation period (7 days, data not shown), we conclude that the sensitivity to exogenous MPA does not appear to be associated with the ability of the mutant fungus to produce MPA and that these are rather independent features. Consequently, our results suggest that, in addition to its role in self-resistance to MPA, *mpaF* also may participate in the production of this compound. Previous kinetic studies suggested that MpaF is an enzyme [24], but the specific reaction that it may catalyze has not been elucidated. The future elucidation of this enzymatic step is necessary to establish the exact role of MpaF in the biosynthetic pathway of MPA in *P*. *roqueforti*.

## Supporting Information

S1 Fig General representation of the plasmids constructed for each gene from the *mpa* cluster. Pgpd and PpcbC represent the convergent promoters from the *gpd* gene from *A*. *nidulans* and the *pcbC* gene from *P*. *chrysogenum*. The grey box represents a fragment of the inserted exon. Seven plasmids were constructed and each one was named pJL-RNAi-mpaX, where X identifies the specific gene (pJL-RNAi-mpaA to pJL-RNAi-mpaH). The size of each insert is indicated in the S1 Table.
(TIF)

S2 Fig (A) Part of the output of a BlastX search using ORF Proq05g069810 as the query. Please note that compared with the rest of the hits, the first hit belonging to CDM36727 (the deduced product of Proq05g069810 itself) lacks the carboxyl end encoded by the last exon, suggesting that Proq05g069810 may span a larger region than that described by the original delimitation of the ORF. (B) Multiple alignment of protein CDM36727 from *P*. *roqueforti* (CDM36727-Pr) and its closer orthologs including the MpaB proteins. Alignment was performed with Clustal Omega using default parameters. Full sequences were aligned, but only the region spanning the carboxyl ends is shown. Please note that the deduced protein CDM36727 is shorter than its orthologs and our independent annotation (named MpaB-Pr). Pr: *P*. *roqueforti*; Pb-NRRL: *P*. *brevicompactum* strain NRRL 864; Pb-IBT: *P*. *brevicompactum* strain IBT 23078; Po: *P*. *oxalicum*; Pe: *P*. *expansum*; Ps: *P*. *solitum*.
(PDF)

S3 Fig (A) Part of the output of a BlastX search using ORF Proq05g069780a as query. According to our analysis, Proq05g069780a has 788 excess nucleotides at the 5´ end. As a consequence, according to BlastX, the first hit (belonging to CDM36724, the deduced product of Proq05g069780a itself) contains two introns (one of them huge) and two additional small exons at the amino end which are not found in its closer homologous proteins. This result suggests that Proq05g069780a may span a shorter region than that described by the original delimitation of the ORF. (B) Multiple alignment of protein CDM36724 from *P*. *roqueforti* (CDM36724-Pr) and its closer orthologs (AndK and MpaDE). Alignment was performed with Clustal Omega using default parameters. Full sequences were aligned, but only the region spanning the amino ends is shown. Please note that the deduced protein CDM36724 is larger than its orthologs and our independent annotation (named MpaDE-Pr). Pr: *P*. *roqueforti*; Pb-NRRL: *P*. *brevicompactum* strain NRRL 864; Pb-IBT: *P*. *brevicompactum* strain IBT 23078; As: *Aspergillus stellatus*.
(PDF)

S4 Fig Result of RACE-PCR experiments performed on the *mpaB* and *mpaDE* genes from *P*. *roqueforti* (A) The 3`end cDNA sequence of *mpaB* obtained by using 3´-RACE-PCR is shown in blue. Above this sequence, the sequence of the deduced intron is shown in black. The arrow indicates the site where the intron interrupts the coding sequence. The stop codon predicted by the original annotation of the gene is underlined into the intron sequence, whereas the stop codon deduced from our experiment is highlighted in a red box. (B) The 5`end cDNA sequence of *mpaDE* obtained by using 5´-RACE-PCR is shown in green. The start codon deduced from our experiment is highlighted by a yellow box. Both in A and B, the results confirmed our proposed delimitation of the genes. In each case, the amplicon obtained from the RACE-PCR procedure was cloned in *E*. *coli* and four independent cDNA clones were sequenced (both strands).
(PDF)

S5 Fig Comparison between the *mpa* clusters of *P*. *roqueforti* and *P*. *brevicompactum* by dot-plot. Above the plot, a simplified scheme of *mpa* cluster is shown. Diagonal continuous lines represent pairwise alignments matching with coding regions. Please note that the intergenic regions do not shown significant alignments, suggesting strong differences in these regions. Pairwise alignments were performed using the BlastN suite-2 sequences (default parameters) and visualized by Blast Dot Matrix Viewer.
(TIF)

S6 Fig (A) Diagrams of the empty expression cassette with a single *Nco*I cloning site in the original silencing vector pJL43-RNAi (left) and the same expression cassette with a fragment of a *mpa* gene (right). The small black arrows represent primers ConfRNAiFW (5´- GCATGCCATTAACCTAGG -3´) and ConfRNAiRV (5´-ACGGTGGCTGAAGATTC -3´), which were used to confirm the integration of the full silencing cassette. In each case, the expected size of each amplicon is shown. (B) PCR assay demonstrating integration of the full silencing cassette in each transformant used in this work. PCR products were subjected to electrophoresis in agarose gels. Lane WT: wild-type strain *P*. *roqueforti* CECT 2905; lane E: *P*. *roqueforti* CECT 2905 containing empty pJL43-RNAi vector; lane S: Standard GeneRuler 1 kb DNA Ladder (Fermentas). Relevant sizes expressed in kb are shown at left.
(TIF)

S7 Fig Representative trace chromatograms at 300 nm for the RNAi-silenced transformants of *P*. *roqueforti*. In each chromatogram, the gene silenced (*mpaA* to *mpaH*) is indicated. For comparison purposes, the chromatogram for the wild-type strain (WT) is also included. All the chromatograms are in the same scale (0.0 to 0.3 units of absorbance and 0 to 25 minutes). Please note that compared with the wild-type strain, the reduction of MPA production in the transformants is drastic.
(TIF)

S8 Fig MPA levels in mycelium and agar from cultures of *P*. *roqueforti*. For a best comparison, data from the *P*. *roqueforti* wild-type (plot A) and RNAi-silenced transformants (plot B) are shown separately. For simplicity, in plot B only one transformant is shown for each gene: T7 (*mpaA*), T7 (*mpaB*), T5 (*mpaC*), T2 (*mpaDE*), T4 (*mpaF*), T3 (*mpaG*) and T5 (*mpaH*). Each *P*. *roqueforti* strain was grown on solid YES medium for 7 days, and MPA was extracted separately from both the mycelium and agar as described in Materials and Methods. Error bars represent the standard deviation of three replicates in three independent experiments. For each strain, no significant differences were found between the level of MPA in the mycelium and the agar (Student’s *t*-test, P < 0.05).
(TIF)

S1 Table Plasmids generated for the RNAi-mediated silencing of the *mpa* genes from *P*. *roqueforti*. The size of the insert ligated in each plasmid and the sequence of the primers used for the amplification of each insert are also included.
(PDF)

S2 Table Sequence of the primers used in qRT-PCR experiments for each *mpa* gene from *P*. *roqueforti*. The name of each primer and the size of the amplicon generated with each pair of primers are also included.
(PDF)

S3 Table Correlation coefficient (R^2^), slope and efficiency of calibration curves obtained for the *mpa* genes from *P*. *roqueforti* analyzed by qRT-PCR (PDF)
