# Supplementary figures and images for: Identification and Functional Analysis of the Mycophenolic Acid Gene Cluster of Penicillium roqueforti
Source: PLoS One. 2016 Jan 11;11(1):e0147047. doi: 10.1371/journal.pone.0147047 (PMC4708987; doi:10.1371/journal.pone.0147047)

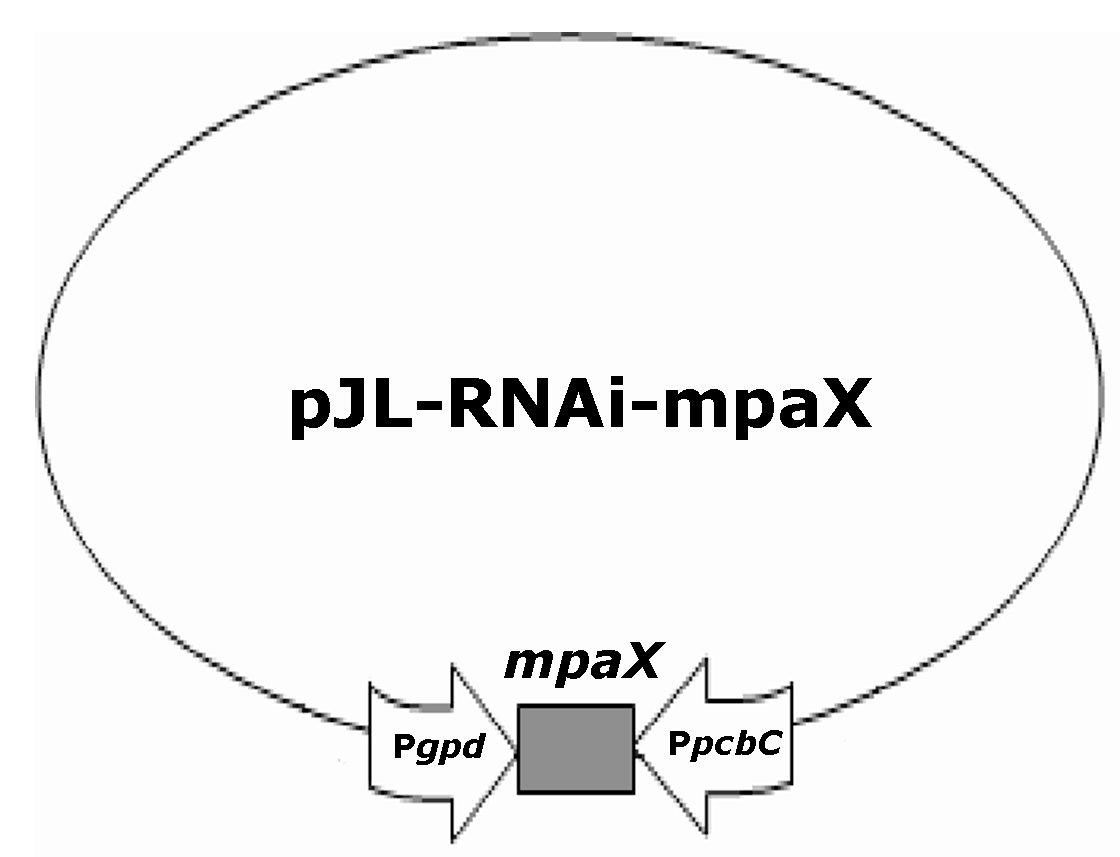

Supplement: S1 Fig — Pgpd and PpcbC represent the convergent promoters from the gpd gene from A. nidulans and the pcbC gene from P. chrysogenum. The grey box represents a fragment of the inserted exon. Seven plasmids were constructed and each one was named pJL-RNAi-mpaX, where X identifies the specific gene (pJL-RNAi-mpaA to pJL-RNAi-mpaH). The size of each insert is indicated in the S1 Table. (TIF) [file pone.0147047.s001.tif]

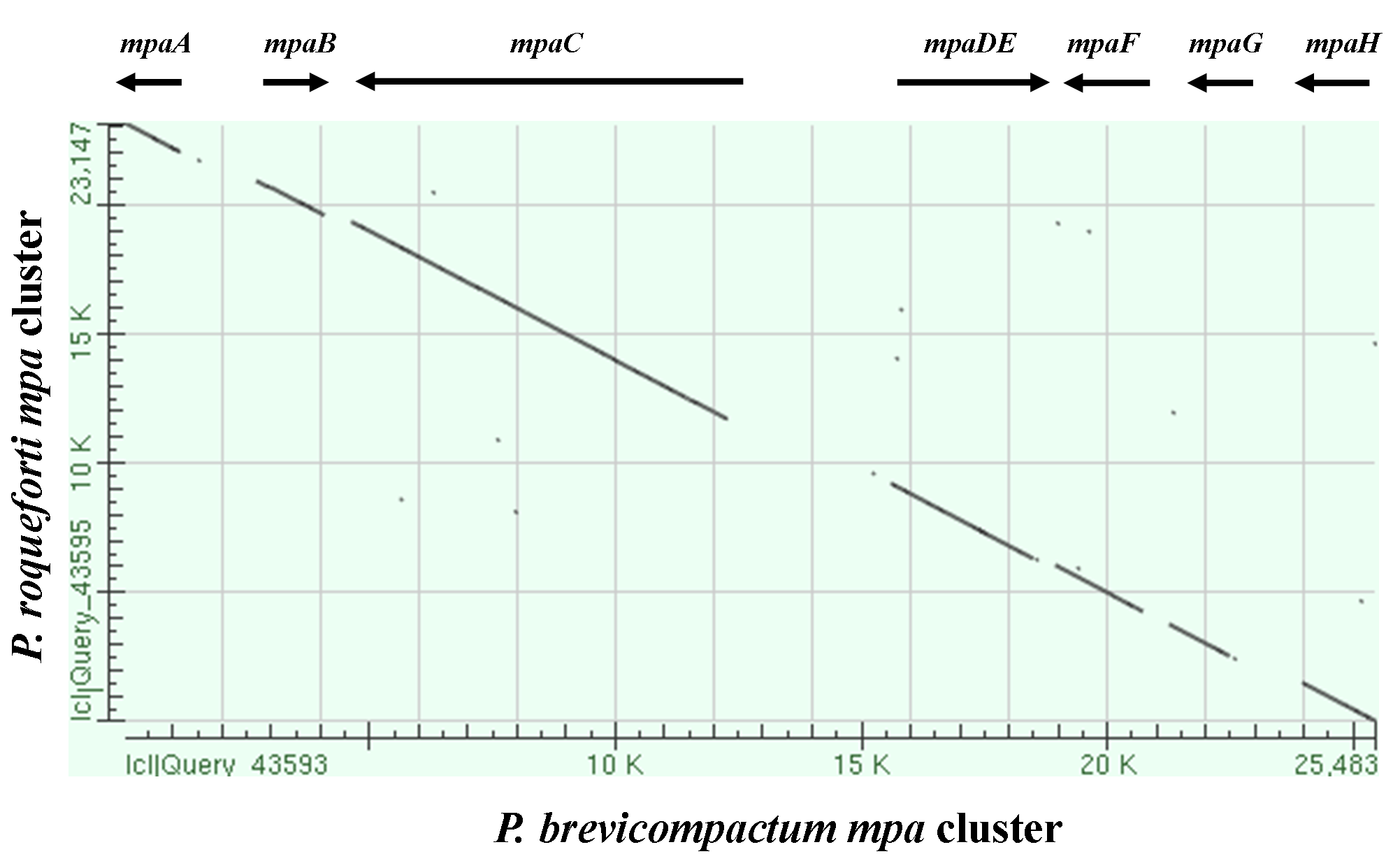

Supplement: S5 Fig — Above the plot, a simplified scheme of mpa cluster is shown. Diagonal continuous lines represent pairwise alignments matching with coding regions. Please note that the intergenic regions do not shown significant alignments, suggesting strong differences in these regions. Pairwise alignments were performed using the BlastN suite-2 sequences (default parameters) and visualized by Blast Dot Matrix Viewer. (TIF) [file pone.0147047.s005.tif]

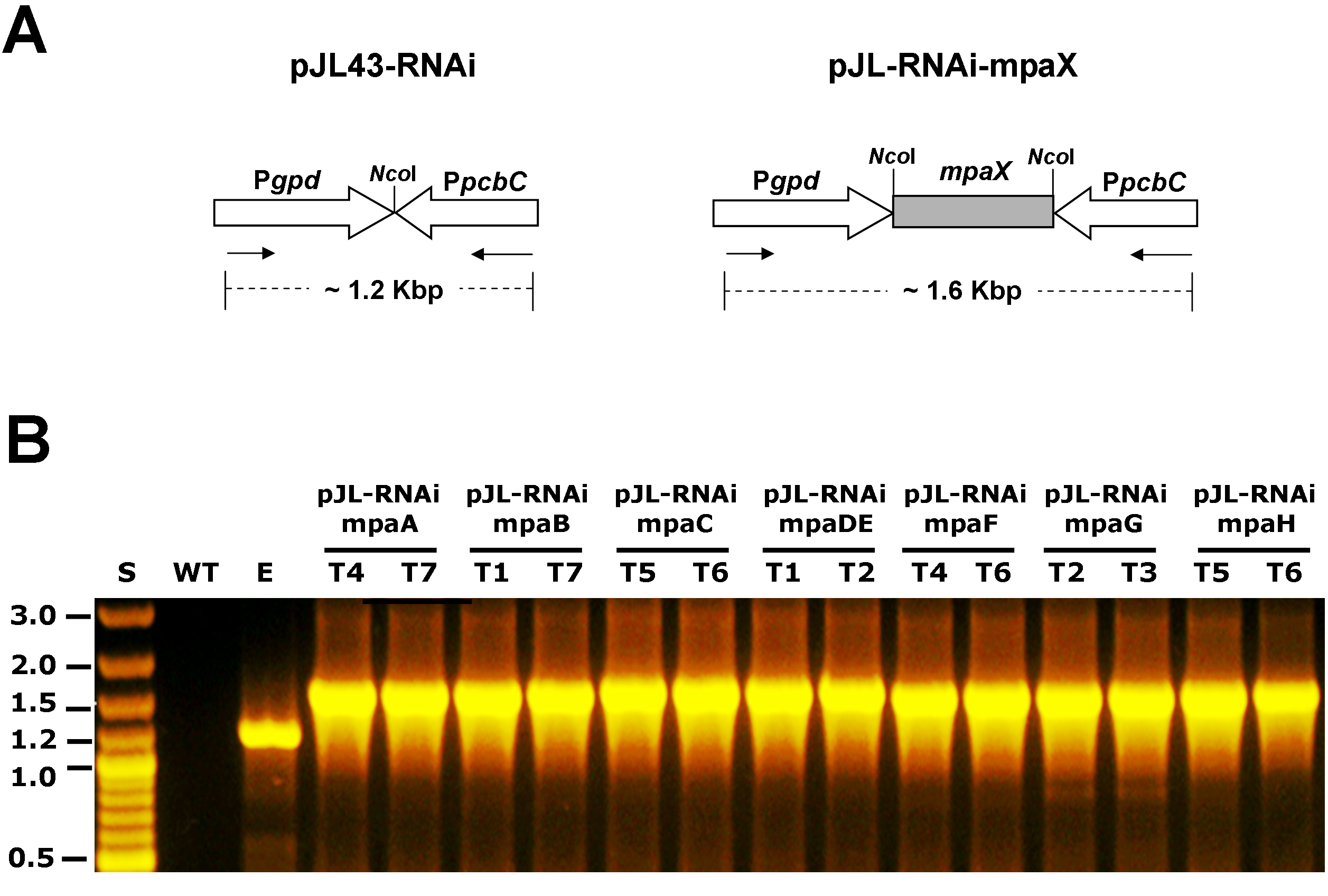

Supplement: S6 Fig — PCR products were subjected to electrophoresis in agarose gels. Lane WT: wild-type strain P. roqueforti CECT 2905; lane E: P. roqueforti CECT 2905 containing empty pJL43-RNAi vector; lane S: Standard GeneRuler 1 kb DNA Ladder (Fermentas). Relevant sizes expressed in kb are shown at left. (TIF) [file pone.0147047.s006.tif]

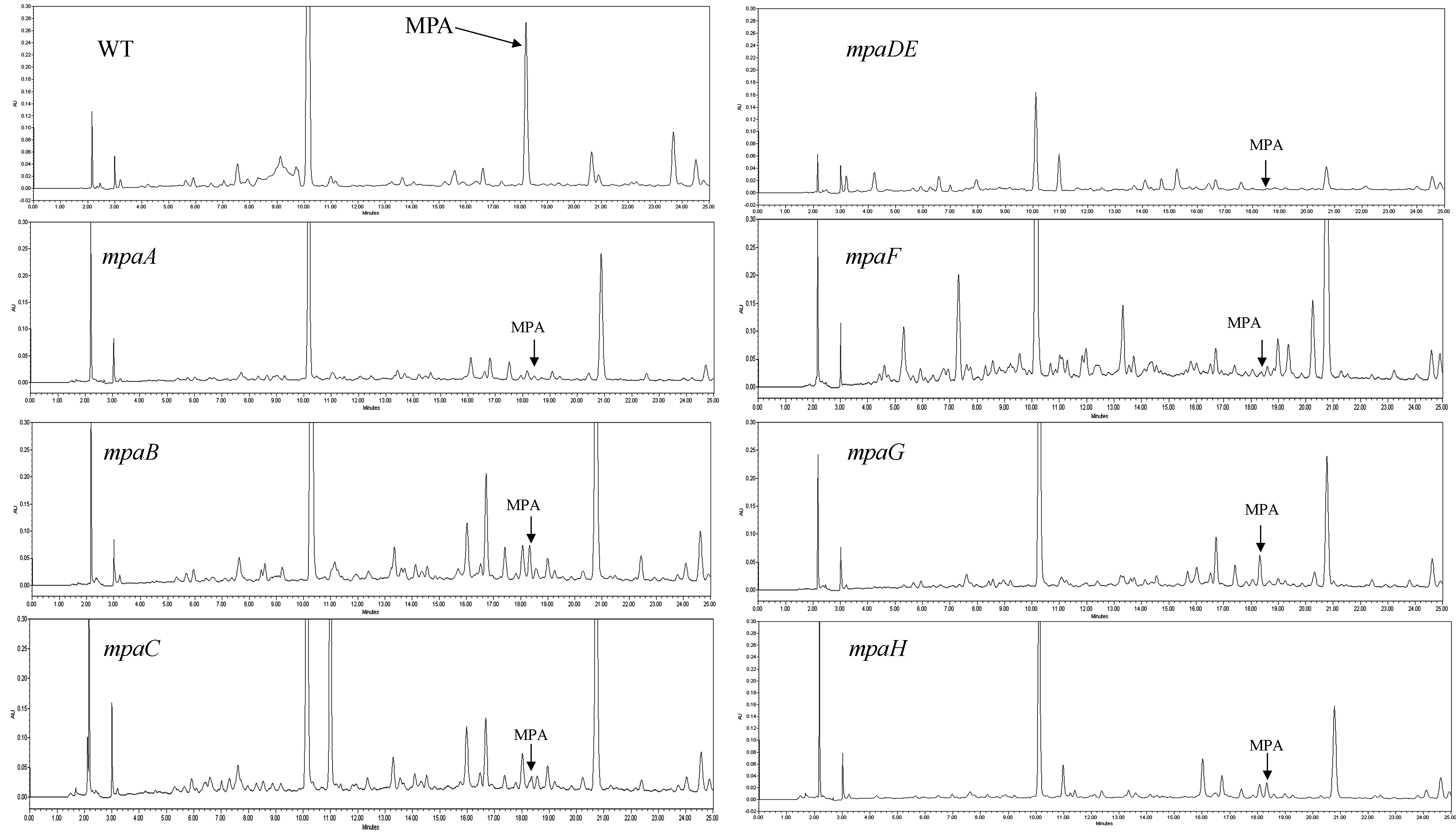

Supplement: S7 Fig — In each chromatogram, the gene silenced (mpaA to mpaH) is indicated. For comparison purposes, the chromatogram for the wild-type strain (WT) is also included. All the chromatograms are in the same scale (0.0 to 0.3 units of absorbance and 0 to 25 minutes). Please note that compared with the wild-type strain, the reduction of MPA production in the transformants is drastic. (TIF) [file pone.0147047.s007.tif]

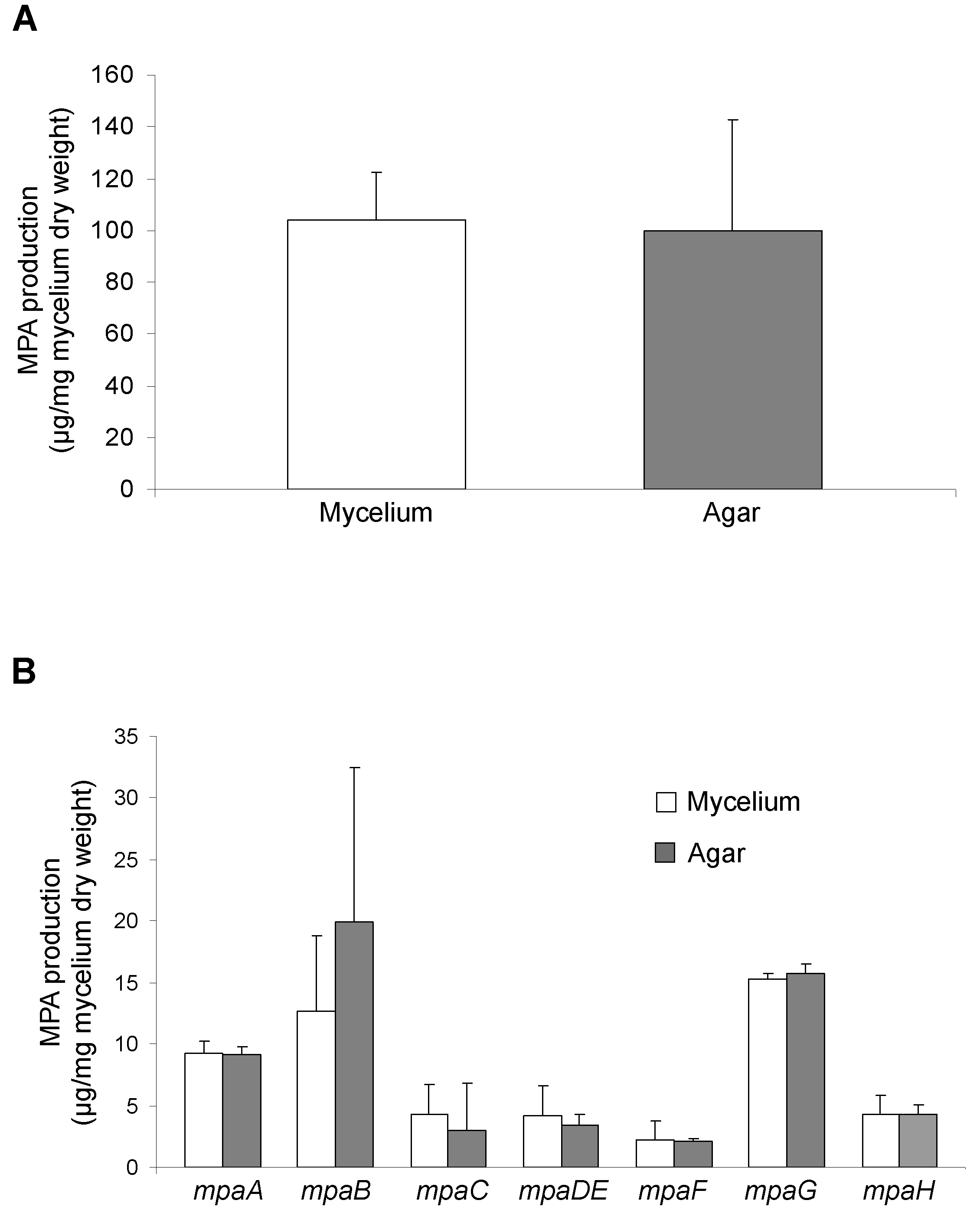

Supplement: S8 Fig — For a best comparison, data from the P. roqueforti wild-type (plot A) and RNAi-silenced transformants (plot B) are shown separately. For simplicity, in plot B only one transformant is shown for each gene: T7 (mpaA), T7 (mpaB), T5 (mpaC), T2 (mpaDE), T4 (mpaF), T3 (mpaG) and T5 (mpaH). Each P. roqueforti strain was grown on solid YES medium for 7 days, and MPA was extracted separately from both the mycelium and agar as described in Materials and Methods. Error bars represent the standard deviation of three replicates in three independent experiments. For each strain, no significant differences were found between the level of MPA in the mycelium and the agar (Student’s t-test, P < 0.05). (TIF) [file pone.0147047.s008.tif]
