# Supplementary material for: Identification and Functional Analysis of the Mycophenolic Acid Gene Cluster of Penicillium roqueforti
Source: PLoS One. 2016 Jan 11;11(1):e0147047. doi: 10.1371/journal.pone.0147047 (PMC4708987; doi:10.1371/journal.pone.0147047)
Supplement: S2 Fig — Alignment was performed with Clustal Omega using default parameters. Full sequences were aligned, but only the region spanning the carboxyl ends is shown. Please note that the deduced protein CDM36727 is shorter than its orthologs and our independent annotation (named MpaB-Pr). Pr: P. roqueforti; Pb-NRRL: P. brevicompactum strain NRRL 864; Pb-IBT: P. brevicompactum strain IBT 23078; Po: P. oxalicum; Pe: P. expansum; Ps: P. solitum. (PDF) [file pone.0147047.s002.pdf]

A)

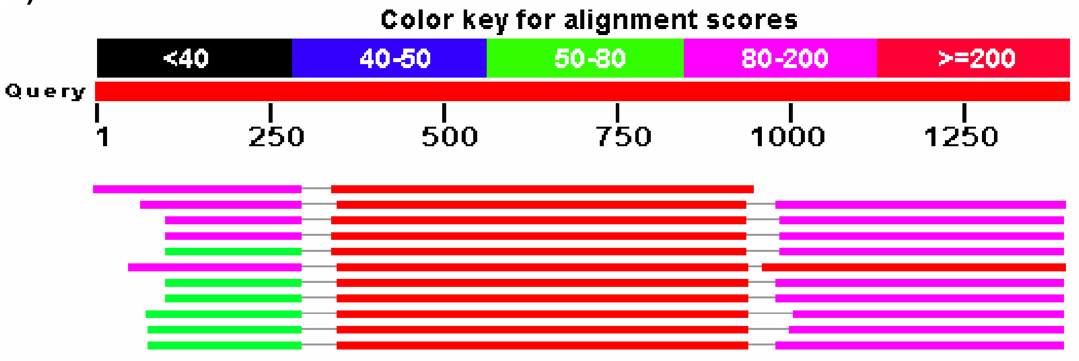

B)

```
CDM36727-Pr        TWSQEYEVKYMVPDAQNRESADQATAVLLYNLPKVFHPVGLQFTSFMMDDRLRKAMLYVE
MpaB-Pr            TWSQEYEVKYMVPDAQNRESADQATAVLLYNLPKVFHPVGLQFTSFMMDDRLRKAMLYEP
MpaB-Pb-NRRL       VWSQDYEAKYMVPDPKNRESADQATAVLLYNLPKILHPIGLQFTSYMMDDRLRKAMLYEA
MpaB-Pb-IBT        VWSQDYEAKYMVPDPKNRESADQATAMLIS-------------------------NRYEA
EPS25740-Po        AWSEAYEAVHMVPDIKNRETADQTTAILLYVLPKVFHPMGLQAVSFMMDDRLRKAMLYDP
KGO42667-Pe        AWSEEYEAKFMVPDAKNRETADQTTAVLVYMLPKMLHPVGLQFVSFMMDDRLRKAMYYDP
KJJ14282-Ps        AWSEEYEAKFMVPDAKNRETADQTTAVLVYMLPKMLHPVGLQVVSFMMDDRLRKAMYYEP

CDM36727-Pr        ------------------------------------------------------------
MpaB-Pr            PTPFWTKVFSALLITRTFILRYLTPPRPLIFAVSNTAKQPDPNNRYYRKSWDALPYYVKP
MpaB-Pb-NRRL       PSPGWSAVFSSLLATRKFVLRYLSPPRPAALAVSNIAQKPDKDDRYHRMSWDALPFYIRP
MpaB-Pb-IBT        PTPGWSMVFSTLLAIRKLILRYLSPPRPAALAVSNIAQKPDKDDRYHRMSWDALPFYIRP
EPS25740-Po        PSPYVSAVMSSILSIRKWVLRYLCLPRPYALRYTPFTEEPDENNRFFLTQWEAAPYYVKP
KGO42667-Pe        PSAFYSALLSTILTARKLFLRYLALPRPYFLRFASFTEEPDQNNRFFLIQWEAAPYYVKP
KJJ14282-Ps        PSAFCAALFSAVLTARKLFLRYLSPPRPYFLRFASFTDEPDENDRFFLTQWEAAPYYVKP

CDM36727-Pr        ------------------------------------------------------------
MpaB-Pr            TFWNRWGPMAWVSWALGHPVPGDQGEKYYPKGYHIHDIGPKYFEGKGQKAIEEMMKELKI
MpaB-Pb-NRRL       TFWNRWGPMAWISWLMAHPVPGDHGQKYYPQGYHIQDIGPKYFEGKGHKEIQEMMKELKI
MpaB-Pb-IBT        TFWNRWGPMAWISWLMGHPVPGDLGQKKGPQGDPGNDEGIKDLKDGEM-SLPLVWSKYHA
EPS25740-Po        TLWNRWGPTALWTRLMGLPVPGDEGEKYYPRGYQLEDVGPKYFEGKGRKSLEVIMEEYKG
KGO42667-Pe        TFWNRWGPMAWLTWALGRPVPGDEGDKYYPTGYSVPDVGPKYFEGKGRKQLDETLLELKE
KJJ14282-Ps        TFWNRWGPMAWLTRALGRPVPGDEGDKYYPNGYSVPDVGPKYFEGKGRKQLDETLSELKG

CDM36727-Pr        ---------
MpaB-Pr            SRTGKCPFH
MpaB-Pb-NRRL       SRTGKCPFH
MpaB-Pb-IBT        TTND-----
EPS25740-Po        YRTGKCPFH
KGO42667-Pe        YRTGKCPFH
KJJ14282-Ps        YRTGKCPFH
```
