# Supplementary material for: Identification and Functional Analysis of the Mycophenolic Acid Gene Cluster of Penicillium roqueforti
Source: PLoS One. 2016 Jan 11;11(1):e0147047. doi: 10.1371/journal.pone.0147047 (PMC4708987; doi:10.1371/journal.pone.0147047)
Supplement: S3 Fig — Alignment was performed with Clustal Omega using default parameters. Full sequences were aligned, but only the region spanning the amino ends is shown. Please note that the deduced protein CDM36724 is larger than its orthologs and our independent annotation (named MpaDE-Pr). Pr: P. roqueforti; Pb-NRRL: P. brevicompactum strain NRRL 864; Pb-IBT: P. brevicompactum strain IBT 23078; As: Aspergillus stellatus. (PDF) [file pone.0147047.s003.pdf]

A)

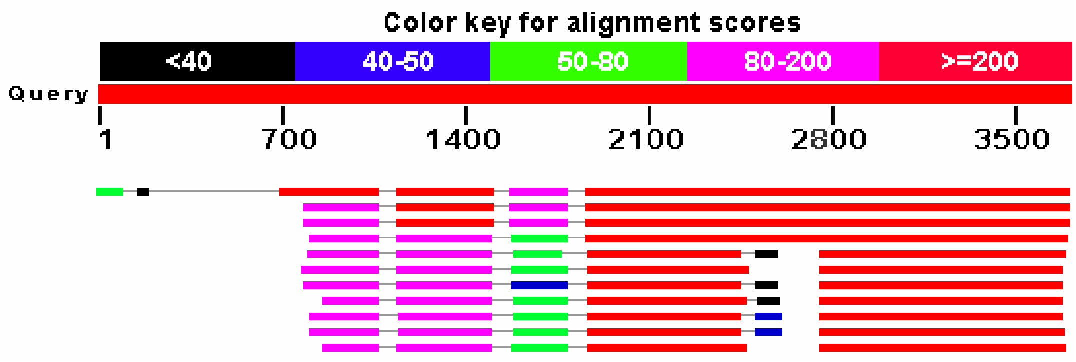

B)

```
AndK-As             ------------------------------------------------------------
CDM36724-Pr         MRVEAGGDMRDKLMWIRLYILGNVGQTFGDMKRYIGMWSGMLFPISTKHTRRLISILKVY
MpaDE-Pr            ------------------------------------------------------------
MpaDE-Pb-NRRL       ------------------------------------------------------------
MpaDE-Pb-IBT        ------------------------------------------------------------

AndK-As             -------------------MMPTVTAVSAILPLAILFVFSRALWKLWYLSDIPGPFWCKL
CDM36724-Pr         FIFILNPPAQVYVQDINLYTMDYLIIIRITAVAVVLYLTRYVCCLYLHLQDVPGPLFAKF
MpaDE-Pr            --------------------MDYLIIIRITAVAVVLYLTRYVCCLYLHLQDVPGPLFAKF
MpaDE-Pb-NRRL       --------------------MESLSLTWITAIAVVLYLVQRYVRSYWRLKDIPGPVLAKL
MpaDE-Pb-IBT        --------------------MKSLSLTWITAVAVVLYLVQRYVRSYWRLKDIPGPVLAKL
```
