# Supplementary material for: Identification and Functional Analysis of the Mycophenolic Acid Gene Cluster of Penicillium roqueforti
Source: PLoS One. 2016 Jan 11;11(1):e0147047. doi: 10.1371/journal.pone.0147047 (PMC4708987; doi:10.1371/journal.pone.0147047)
Supplement: S4 Fig — The start codon deduced from our experiment is highlighted by a yellow box. Both in A and B, the results confirmed our proposed delimitation of the genes. In each case, the amplicon obtained from the RACE-PCR procedure was cloned in E. coli and four independent cDNA clones were sequenced (both strands). (PDF) [file pone.0147047.s004.pdf]

A)

GTATGTGGAATAGTAAGATCTGATCTTATAAAACGAAATATCGAGCGCTAACATCAACTAG

GTGGGATTGCAGTTCACTTCGTTCATGATGGATGACCGGCTGAGGAGGGCTATGTTATACG
AACCACCAACGCCATTCTGGACAAAAGTCTTCTCTGCCCTCCTAATAACCCGCACATTCAT
TCTCCGCTACCTAACACCCCCACGACCTTTGATCTTCGCGGTGTCAAATACAGCCAAGCAA
CCCGATCCAAACAACCGCTATTACCGCAAATCTTGGGACGCACTTCCCTACTACGTGAAAC
CAACATTTTGGAATAGATGGGGTCCAATGGCGTGGGTTTCCTGGGCGCTCGGTCATCCTGT
CCCGGGCGACCAGGGTGAGAAGTACTATCCGAAGGGGTATCATATTCACGATATCGGGCCG
AAGTATTTTGAAGGAAAGGGGCAGAAGGCAATTGAGGAGATGATGAAGGAACTGAAGATTT
CTAGGACGGGAAAGTGTCCTTTTCATTAGTTTAAAGCAAGTCTTATGATTTAGTGAAGTTC
AGACCGCATGGATACTTGGAGGAAAAAAAAAAAAAAAAAAAAAAAAAAAAAAAAAAAAAAA
AAA

B)

TCCTCCAGCACAAGTATACGTACAAGACATAAACTTATATACTATGGACTATCTGATCATA
ATACGTATCACAGCTGTTGCTGTTGTGCTATATCTCACCCGCTACGTTTGTTGTCTATACT
TGCATCTGCAAGATGTACCAGGTCCGTTGTTTGCAAAGTTCACAAACCTGCAACG
