# Supplementary material for: Identification and Functional Analysis of the Mycophenolic Acid Gene Cluster of Penicillium roqueforti
Source: PLoS One. 2016 Jan 11;11(1):e0147047. doi: 10.1371/journal.pone.0147047 (PMC4708987; doi:10.1371/journal.pone.0147047)
Supplement: S1 Table — The size of the insert ligated in each plasmid and the sequence of the primers used for the amplification of each insert are also included. (PDF) [file pone.0147047.s009.pdf]

S1 Table. Plasmids generated for the RNAi-mediated silencing of the *mpa* genes from *P. roqueforti*. The size of the insert ligated in each plasmid and the sequence of the primers used for the amplification of each insert are also included.

Plasmid	Size of the insert (bp)	Sequence of primers (5`--3`) used in amplification of inserts*
pJL-RNAi-mpaA	303	Fw: AGACTCCCATGGGCACCCCGATCTGCCTTC
		Rv: AGACTCCCATGGAATGAATACGCCCGCCAC
pJL-RNAi-mpaB	354	Fw: AGACTCCCATGGCCTCCTTCAAGCGCTATA
		Rv: AGACTCCCATGGTATCCACCTCCTCGAGCC
pJL-RNAi-mpaC	368	Fw: AGACTCCCATGGCCAGATAGTGAAGCAGAA
		Rv: AGACTCCCATGGGTCAAGGTCTGTTCAAGA
pJL-RNAi-mpaDE	372	Fw: AGACTCCCATGGCATGATCAAATCACAGAC
		Rv: AGACTCCCATGGGCGGCATTTAGAAGATAG
pJL-RNAi-mpaF	348	Fw: AGACTCCCATGGACCGGCAGGCACTACTCT
		Rv: AGACTCCCATGGATATTACCGCCGATAACG
pJL-RNAi-mpaG	384	Fw: AGACTCCCATGGCCGACGCTGAGTGGAACT
		Rv: AGACTCCCATGGCGGTGTGAAGATGTTATG
pJL-RNAi-mpaH	357	Fw: AGACTCCCATGGTCCTCGGTGCGTAGATCG
		Rv: AGACTCCCATGGACATTCAAAAACGTCGCC

* *Nco*I sites are highlighted in red.
