# Supplementary material for: Identification and Functional Analysis of the Mycophenolic Acid Gene Cluster of Penicillium roqueforti
Source: PLoS One. 2016 Jan 11;11(1):e0147047. doi: 10.1371/journal.pone.0147047 (PMC4708987; doi:10.1371/journal.pone.0147047)
Supplement: S2 Table — The name of each primer and the size of the amplicon generated with each pair of primers are also included. (PDF) [file pone.0147047.s010.pdf]

S2 Table. Sequence of the primers used in qRT-PCR experiments for each *mpa* gene from *P. roqueforti.* The name of each primer and the size of the amplicon generated with each pair of primers are also included.

Target gene	Name of the primer	Sequence (5`--3`)	Amplicon size (bp)
mpaA	mpaA-qPCR-FW	ATTGTCGGAATCAGCCCATA	118
	mpaA-qPCR-RV	CCAGCCTCTTACGGTCTTCA	
mpaB	mpaB-qPCR-FW	TTTGGAACGCTGTGCTATTG	98
	mpaB-qPCR-RV	CCCTAAACCCCGTCTTACCT	
mpaC	mpaC-qPCR- FW	CAGGGGTTCTGTGTGGGTAT	108
	mpaC-qPCR-RV	AATACAGACAGCGAGCCGTA	
mpaDE	mpaDE qPCR- FW	GACAGAAAAGGAAGCCATCA	87
	mpaDE qPCR- RV	CCTGCGGCATTTAGAAGATA	
mpaF	mpaF-qPCR-FW	ATGGAACAGTTCGCTTTGAG	85
	mpaF-qPCR-RV	AACTTCTTCTCGTGGGTGTG	
mpaG	mpaG-qPCR-FW	CGGGTAAGGGGATAGATTGT	99
	mpaG-qPCR-RV	TCACATTCATAGCCACGAGA	
mpaH	mpaH-qPCR-FW	CCGCTGATACTACTGCCACT	85
	mpaH-qPCR-RV	GCATTGAAGTTCTGCCGTAT	
β-tubulin	qRT-btub-fw	TCCAAGGTTTCCAGATCACC	89
	qRT-btub-rv	GAACTCCTCACGGATCTTGG	
