# Supplementary material for: Identification and Functional Analysis of the Mycophenolic Acid Gene Cluster of Penicillium roqueforti
Source: PLoS One. 2016 Jan 11;11(1):e0147047. doi: 10.1371/journal.pone.0147047 (PMC4708987; doi:10.1371/journal.pone.0147047)
Supplement: S3 Table — (PDF) [file pone.0147047.s011.pdf]

S3 Table. Correlation coefficient (R^2), slope and efficiency of calibration curves obtained for the *mpa* genes from *P. roqueforti* analyzed by qRT-PCR

	β-tubulin	*mpaA*	*mpaB*	*mpaC*	*mpaDE*	*mpaF*	*mpaG*	*mpaH*
R^2	0.980	0.996	0.997	0.995	0.993	0.994	0.994	0.996
Slope	-3.331	-3.561	-3.450	-3.448	-3.583	-3.192	-3.335	-3.189
Efficiency (%)	99.635	90.913	94.912	94.995	90.157	105.714	99.449	105.844
